# The Increased Risk of Hypertension Caused by Irrational Dietary Pattern May Be Associated with Th17 Cell in the Middle-Aged and Elderly Rural Residents of Beijing City, Northern China: A 1:1 Matched Case-Control Study

**DOI:** 10.3390/nu15020290

**Published:** 2023-01-06

**Authors:** Cheng Li, Yaru Li, Nan Wang, Zhiwen Ge, Zhengli Shi, Jia Wang, Bingjie Ding, Yanxia Bi, Yuxia Wang, Yisi Wang, Zhongxin Hong

**Affiliations:** Department of Clinical Nutrition, Beijing Friendship Hospital, Capital Medical University, 95 Yongan Road, Xicheng District, Beijing 100050, China

**Keywords:** hypertension, dietary pattern, immune dysfunction, CD4+ T cells, middle-aged and elderly people

## Abstract

An irrational diet has been widely considered as one of the vital risk factors of hypertension. Previous studies have indicated that immune dysfunction may be involved in the pathogenic process of hypertension, while fewer studies have mentioned whether CD4+ T cells are involved in the association between dietary pattern and hypertension. This present 1:1 matched case-control study was conducted to analyze the association among dietary pattern, CD4+ T cells and hypertension. A total of 56 patients with diagnosed hypertension and 56 subjects without diagnosed hypertension in the rural area of Beijing City, northern China, were matched by age and gender, and then classified into a case group and a control group, respectively. Compared with the control group, higher frequencies of pro-inflammatory CD4+ T cells, such as Th1, Th1(IFN-γ), Th17(IL-17A), and Th1/17 (IFN-γ/IL-17A), were found in the case group (*p* < 0.05). A significantly higher level of circulating IL-17A was also found in the case group (7.4 pg/mL vs. 8.2 pg/mL, *p* < 0.05). Five dietary patterns were identified using exploratory factor analysis. An irrational dietary pattern, characterized by high-factor loadings of refined wheat (0.65), meat (0.78), poultry (0.76), and alcoholic beverage (0.73), was positively associated with SBP (*β* = 5.38, 95%*CI* = 0.73~10.03, *p* < 0.05) in the multiple linear regression model with the adjustment of potential covariates. The other dietary patterns showed no significant association with blood pressure. Furthermore, meat, processed meat, and animal viscera were positively correlated with the peripheral Th17 or Th1/17. In conclusion, the irrational dietary pattern characterized by refined wheat, meat, poultry, and alcoholic beverage, was positively correlated with blood pressure, and may increase the risk of hypertension in the rural area of Beijing, northern China. Th17, a subset of the CD4+ T helper cells, may be involved in the association between irrational dietary pattern and hypertension.

## 1. Introduction

Hypertension has been widely recognized as a major risk factor for cardiovascular disease and has been drawing a worldwide public health concern [1,2]. With the socio-economic development and the alteration of dietary structure in recent decades, the prevalence of hypertension has been rapidly increasing in China [3,4]. According to the data from a large hypertension survey including 31 provinces in mainland China, about 23.2% of Chinese adults suffered hypertension and 41.3% adult suffered pre-hypertension [5]. In addition, another large sample size cross-sectional study in northern China, where residents typically characterized by higher consumption of dietary salt, animal food, and alcohol consumption [6,7], reported that about 55.7% of participants had hypertension [8]. Therefore, there is an imperative and urgent need to alter the unhealthy dietary structure and control blood pressure in China, especially for the residents in the rural area of northern China.

In the past decade, increasing scientific evidence has suggested that the adoption of a healthy dietary pattern, such as the Dietary Approaches to Stop Hypertension (DASH), may be helpful for the prevention and management of hypertension [9]. Furthermore, the geographical dietary pattern in southern China was also proven to be associated with lower blood pressure [10] and was recommended as a healthy dietary pattern for the prevention of non-communicable diseases (NCDs) [11]. Generally, the Jiangnan dietary pattern could be characterized by a high consumption of fresh vegetables and fruits in season, fresh water fish, shrimp, legumes; a moderate consumption of whole-grain rice, plant oils, and red meat; in addition, a relatively low consumption of salt and millet wine [11]. However, dietary patterns vary depending on the local environment, climate, and food availability [11,12]. For residents in northern China, especially those living in the rural area, recognizing and then altering the irrational dietary factors for hypertension may be more valuable and much easier to be implemented.

At present, relevant studies about the potential mechanism between dietary nutrition and hypertension have been focused more on the specific dietary nutrients, such as sodium [13] and magnesium [14]. Although many epidemiological studies have presented the intense relationship between the rational dietary patterns and hypertension, the underlying mechanism is still unclear. Previous studies indicated that the dysfunctional immune response and systematically chronic inflammation are triggers for hypertension [15]. The composition and distribution of different subsets of CD4+ T cells have been proven to be crucial for vascular function and blood pressure [16,17,18]. Animal studies suggested that high fat or high salt consumption could increase the levels of pro-inflammatory CD4+ T cells and exacerbate chronic inflammation [19,20]. Dietary fiber and its intestinal metabolites, such as short chain fatty acids, may be protective for inflammation and vascular integrity [21,22]. Traditional dietary patterns in northern China, especially in the rural area, were generally characterized by a high consumption of plant-derived food and salt, and a low consumption of fresh fruits, seafood, and milk [10,11,23]. Because of the complexed dietary habit, the association between dietary pattern and hypertension are still unclear in northern China. Furthermore, the potential mechanism between dietary pattern and hypertension has not been fully understood.

Based on previous studies, we hypothesized that the geographical dietary pattern may be associated with hypertension in northern China. Therefore, the 1:1 matched case-control study was conducted in a rural community of Beijing City, to explore the association between dietary pattern and hypertension in middle-aged and elderly rural residents. Furthermore, subsets of CD4+ T cells in peripheral blood were also detected and compared between participants with and without hypertension. The relationship between dietary factors and subsets of CD4+ T cells was also analyzed to explore the potential mechanism between the whole diet and hypertension.

## 2. Materials and Methods

### 2.1. Participants and Study Desgin

This present 1:1 matched case-control study was conducted in a rural community in Fangshan district, Beijing City, in 2021. About 60 patients aged above 45 years with a definite record of hypertension diagnosis [24], according to the electronic medical record of local community hospital, were included in the present study. Controls were recruited from the rural residents in the same community and matched to cases by gender and age. Individuals with the following conditions were excluded: (1) autoimmune disease; (2) mentally incompetent; (3) severe liver or renal disease; (4) disability or obstacle for communication or investigation. A total of 56 cases and 56 controls finally completed the study and were analyzed in the present study. This study was conducted in accordance with the Declaration of Helsinki and was approved by the Ethics Committee of Beijing Friendship Hospital, Capital Medical University (Ethics number: 2021-P2-223-01).

### 2.2. Anthoropometric Measurments

All the anthropometric measurements were conducted in the field by trained doctors and clinical dietitians. Body weight (kg) and height (m) were measured at once, respectively. Body mass index (BMI) was calculated as weight (kg) divided by height (m) squared. On-site blood pressure, including systolic blood pressure (SBP) and diastolic blood pressure (DBP), was measured twice in a quiet sitting position, using an electronic sphygmomanometer (Omron HEM-907, Omron Healthcare, Kyoto, Japan). The average value of the two measurements was used for analysis. Handgrip strength was then assessed in a standing position using an electronic handheld dynamometer (CAMRY EH101, Xiangshan, Zhongshan, Guangdong, China). Both hands were, respectively, assessed, once, and the maximum HGS was used for analysis. Waist circumference (WC, cm) and hip circumference (HC, cm) were all measured in a standing position by the trained investigators using a non-elastic tape.

### 2.3. Questionnarie and Food Consumption Assessment

Age, gender, and hypertension diagnosis information were collected from the electronic medical record of the local community hospital at the beginning of the study and were rechecked by an investigator in the face-to-face interview. Lifestyle and daily exercise were reported by participant and then recorded in the electronic questionnaire by a trained investigator in the face-to-face interview. Type 2 diabetes (T2Ds), chronic kidney disease (CKD), gout, and hyperlipidemia with define diagnosis were recorded. Relevant medication history was also recorded by investigators. A 67-item, semi-quantitative food frequency questionnaire (FFQ) was used for food consumption assessment. Food items were designed by clinical dietitians based on previous surveys in the community to match local dietary habit. Food consumption frequency (yearly, monthly, weekly, daily) and usual consumption amount were the two main parts of the FFQ. Food consumption frequency for each food item was evaluated and reported by the participants. The usual consumption amount and the portion size of each food were explained to the participants by clinical dietitians using a food atlas and a 1:1 real food model, one by one. Daily consumption of each food item was calculated according to the food consumption amount and consumption frequency.

### 2.4. CD4+ T-Cell Subset and Inflammatory Cytokines

Heparin anti-coagulated peripheral blood samples were collected by doctors in the morning after a 12 h fast. The distribution of the circulating T-cell subset in peripheral blood samples was determined by flow cytometry. T helper cells were identified by different expression of CCR6 and CXCR3. Naïve CD4 T cell (CD4+CD45RA+; Th0), T helper 1 cell (CD4+CD45RA-CXCR3+CCR6-, Th1), T helper 2 cell (CD4+CD45RA-CXCR3-CCR6-, Th2), T helper 17 cell (CD4+CD45RA-CXCR3-CCR6+, Th17), and T helper 1/17 cell (CD4+CD45RA-CXCR3+CCR6+, Th1/17) were respectively analyzed as subsets of T helper cells in peripheral blood. In addition, the intracellular expression of inflammatory cytokines in CD4+ T cells, including interferon-γ (IFN-γ), interleukin-4 (IL-4), and interleukin-17A (IL-17A), were also detected for the determination of phenotypes of T helper cells. CD4+ T helper cells were characterized by Th1 (IFN-γ), Th2 (IL-4), Th17 (IL-17A), and Th1/17 (IFN-γ/ IL-17A) by the intracellular cytokines, respectively. The T regulator cell (Treg) was identified by the expression of fork head box P3 (Foxp3) in CD4+ T cells. Circulating inflammatory cytokines, including IL-6, IL-10, and IL-17A, were respectively detected by ELISA.

### 2.5. Dietary Pattern Analysis

A total of 67 items of food in the FFQ were merged into 24 food groups according to food type and daily consumption for the analysis of dietary pattern. Exploratory factor analysis was used to extract dietary patterns based on the 24 food groups. Dietary patterns were identified and retained based on the scree plot (a plot of the eigenvalues of principal components) and the interpretability [25]. Each pattern was named with the food groups with the highest loading. A factor score of each dietary pattern was calculated by the consumption amount of each food group weighted by their factor loading [25]. A higher pattern score reflects closer adherence to the identified dietary pattern.

### 2.6. Stastistical Analysis

Quantitative data were presented as medians and interquartile ranges (Q1, Q3). Comparisons between the two groups were performed by paired t-test for normally distributed variables, or Wilcoxon signed-rank test for non-normally distributed variables. Shapiro–Wilk test and Q-Q plot were used for the test of normal distribution. Categorical data were presented as number and percentage; comparisons between groups were performed by chi-square test or Fisher’s exact test. Exploratory factor analysis was conducted for the identification of dietary patterns by *proc factor* step in SAS 9.4 system (SAS Institute, Cary, NC, USA). Matrix factor loading, explained variance and score of each dietary pattern, were all outputted by the system. A multiple linear regression model was used to analyze the association between the score of each dietary pattern and on-site blood pressure. Model 1 was an unadjusted model; Model 2 was adjusted by age, gender, BMI, group, antihypertensive drug use, daily exercise level, T2Ds, CKD, gout, hyperlipidemia, smoke status, drink status, and self-reported dietary habits (salt and fat). A heatmap was created to analyze the correlation between the subset of CD4+ T cells, and consumption of each food group, *p* < 0.1, was marked in the heatmap. In addition, dietary patterns and circulating inflammatory cytokines were also involved in the analysis. All heatmaps were created with the R package *pheatmap* (v1.0.12) in R project (v4.0.2, R Foundation, Vienna, Austria). *p* < 0.05 was recognized as statistically significant in the present study.

## 3. Results

### 3.1. General Characteristics

General characteristics of all 112 participants are presented in Table 1. The participants in the case group and the control group were 1:1 matched by gender and age, respectively. There was no significant difference between the two group regarding the measured anthropometric indictors (*p* > 0.05), including BMI, WC, HC, and HGS. Meanwhile, the difference of on-site blood pressure between the two groups remained statistically insignificant (*p* > 0.05). Compared with the control group, self-reported daily salt consumption in the case group was significantly lower (*p* = 0.040). A total of 42.9% (24/56) participants with hypertension reported light salt consumption, while only 23.2% (13/56) participants without hypertension reported light salt consumption. The differences of the other lifestyle entries, including daily exercise, daily fat consumption, drinking, and smoking, all remained insignificant between the two group (*p* > 0.05). In addition, the prevalence of T2Ds, CKD, gout, and hyperlipidemia between cases and controls also showed no significant difference.

### 3.2. Comparisons of CD4+ T-Cell Subsets in Participants with and without Hypertension

The differences of CD4+T-cell subsets in both forms of absolute number (1000/mL) and frequency (%) were compared between the two matched groups (Table 2). Compared with the control group, the frequency of Th0 in the case group was significantly lower (32.0 vs. 27.3, *p* < 0.01), while no significant difference of the absolute number of Th0 between the two groups was observed (280.5 vs. 210.6, *p* > 0.05). Adversely, the frequency of Th1 in the control group was significantly lower than the case group (20.6 vs. 24.5, *p* < 0.01), and the absolute number of Th1 in the control group was also significantly lower than Th1 in the case group (171.3 vs. 220.6, *p* < 0.01). There was no significant difference of the other T helper cells (Th2, Th17, Th1/17) between the two groups. In addition, intracellular cytokines (IFN-γ, IL-4, and IL-17A) in CD4+ T cells between the two groups were also compared. Compared with the control group, the frequency of Th1 (IFN-γ) in CD4+ T cells in the case group was significantly higher (18.9 vs. 21.1, *p* < 0.01), while no significant difference of the absolute number of Th1 (IFN-γ) between the two groups was observed (147.0 vs. 174.4, *p* > 0.05). Th2 (IL-4) and Th17(IL-17A) showed no significant difference between the two groups (*p* > 0.05). The frequency and absolute number of peripheral Th1/17(IFN-γ/IL-17A) cell and Treg cell in the control group were both significantly lower than the case group (*p* < 0.05).

### 3.3. Comparisons of Circulating Inflammatory Cytokines in Participants with and without Hypertension

Furthermore, circulating inflammatory markers, including IL-6, IL-10, and IL-17A, were also detected and compared between the two groups (Table 3). No significant difference of IL-10 was observed between the two groups. The level of IL-6 in the control group was significantly higher than the case group (1.8 vs. 1.0, *p* < 0.05), while the level of IL-17A in the control group was significantly lower than the case group (7.4 vs. 8.2, *p* < 0.05).

### 3.4. Identified Dietary Patterns

Five dietary patterns were identified from the 112 participants by exploratory factor analysis (Table 4). Matrix factor loadings of those food groups were presented in the five identified dietary patterns, respectively. According to the highest value of matrix factor loadings, five dietary patterns were respectively named as DP1 for “meat-poultry-alcoholic beverage” pattern, DP2 for “legume-soybean-tuber” pattern, DP3 for “fruit-processed meat” pattern, DP4 for “fried cereal-soft drink” pattern, and DP5 for “animal viscera-snack” pattern. DP1 explained 10.90% variance of all the food consumption in the participants, DP2 explained 10.12%, DP3 explained 7.12%, DP4 explained 6.97%, and DP5 explained 6.39%. The five identified dietary patterns explained 41.50% variance of all the food consumption of the 112 participants.

### 3.5. Association between Dietary Pattern and On-Site Blood Pressure

A multiple linear regression model was conducted to analyze the association between identified dietary pattern and on-site blood pressure (Table 5). Factor scores of all five dietary patterns were calculated and analyzed in this present study. In the unadjusted model, DP1 showed a significantly positive association with SBP (*β* = 3.96, 95%*CI* = 0.25~7.66, *p* < 0.05). In addition, with the adjustment of the potential covariates, the positive association between the B pattern and SBP remained statistically significant (*β* = 5.38, 95%*CI* = 0.73~10.33, *p* < 0.05). The other three identified dietary patterns showed no significant association with SBP (*p* > 0.05). In addition, none of the five identified dietary patterns significantly associated with DBP in this present study (*p* > 0.05).

### 3.6. Correlation between Food Consumption and the Subset of CD4+ T Cells

**Figure 1:**
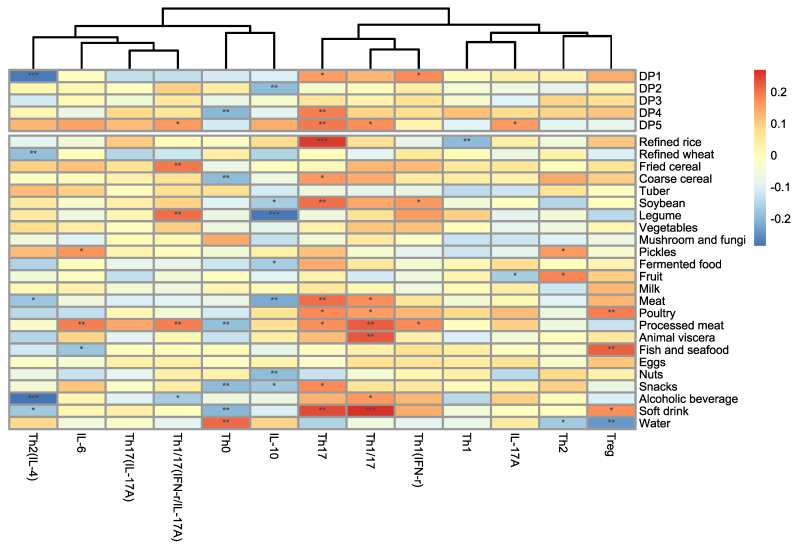
Correlation between food group and the subset (%) of CD4+ T cells. Correlation coefficient *r* > 0 was colored by red; *r* < 0 was colored by blue, * *p* < 0.1, ** *p* < 0.05, *** *p* < 0.01.

Correlations of food groups and of CD4+ T cells (%) were calculated and shown in a heatmap (Figure 1). In addition, the identified DPs and circulating inflammatory cytokines were also analyzed. Correlations of food groups and of CD4+ subsets (absolute number) were calculated and were presented in supplementary Figure S1. The significantly negative correlation was observed between DP1 and Th2 (IL-4). DP4 and DP5 both showed significantly positive correlation with Th17. DP4 also showed significantly negative correlation with Th0. Compared with the other CD4+ T cells, Th17 showed the most significantly positive correlations with multiple food groups, and Th0 showed the most significantly negative correlations with multiple food groups. Soft drink, animal foods, including meat, processed meat, and animal viscera, showed positive correlations with Th17 or Th1/17. Fired cereal, legume, and processed meat all showed significantly positive correlations with Th1/17 (IFN-γ/IL-17A). Th2 (IL-4) showed significantly negative correlation with refined wheat and alcoholic beverage. Poultry, fish, and seafood showed significantly positive correlation with Treg, while water showed significantly negative correlation with Treg. Processed meat showed significantly positive correlation with IL-6. Meat, nuts, and legume showed significantly negative correlation with IL-10, respectively.

## 4. Discussion

An irrational diet has been recognized as a risk factor for hypertension, while, the underlying mechanism remains unclear. Immune dysfunction, inflammation, and hypertension were proven to be intensively related to each other [15]. Effector T cells and regulatory lymphocytes, part of the adaptive immune system, have been proven to be vital triggers for blood vessels constriction and hypertension in recent years [16,17,18]. Several dietary patterns have been recommended for prevention and management of hypertension and relevant NCDs [9,10,11]. However, to the best of our knowledge, fewer of the previous population-based studies discussed the association between dietary pattern and effector T cells in the pathogenesis of hypertension. In addition, those rational dietary patterns were difficult to be recommended for the residents in rural areas of northern China, mainly due to the different climate, agricultural production, traditional habits, and the other relevant reasons.

In this present 1:1 matched case-control study, five dietary patterns were identified. DP1, characterized by the preferred consumption of refined wheat, meat, poultry, and alcoholic beverage, was positively associated with SBP in the multiple linear regression model. However, none of the five identified dietary patterns was conducive to the prevention and management of hypertension. This result also reflected the poor quality of the whole diet in this area. Furthermore, compared with the control group, higher levels of pro-inflammatory subsets of CD4+ T cells were found in the case group. In addition, soft drink and animal foods were positively correlated with pro-inflammatory Th17 and Th1/17, while DP1 was inversely correlated with the anti-inflammatory Th2 (IL-4). In conclusion, the irrational dietary pattern was positively associated with blood pressure and may increase the risk for hypertension in rural residents. Reducing the consumption of animal foods, alcoholic beverage, and soft drink, may help to improve the whole dietary quality, and control hypertension in the rural area of northern China. This present study partially expounded the association among daily diet, immune system, and hypertension. The relevant results provided scientific evidence for the prevention and management of hypertension, especially for the rural residents in northern China with irrational dietary habits.

### 4.1. Hypertension and CD4+ T Cells

In recent years, imbalance of T lymphocytes has been recognized as one of the risk factors for hypertension. According to the production of specific cytokines, CD4+ T cells could be classified into four categories: Th1 cells (IFN-γ), Th2 cells (IL-4 and IL-13), Th17 (IL-17A), and Treg cells (Foxp3 and the secretion of IL-10). Th1 and Th17 cells could promote pro-inflammatory responses by stimulating M1-marcophagtes, whereas Th2 and Treg cells were correlated with anti-inflammatory responses. The differentiation of Th0 could be activated by hypertensive stimuli via the central nervous system and APC [26,27]. Pro-inflammatory Th1 and T17 then triggered inflammation and oxidative stress, and caused vascular damage and the increase of blood pressure [28]. In addition, aldosterone, a mineralocorticoid hormone that controls body fluid and electrolyte balance, has been proven to be involved in the association between immune cell activation and hypertension [29]. Excess aldosterone was pathologically associated with essential hypertension and increased blood pressure [29]. Aldosterone could also induce the activation of dendritic cells and increase the polarization of Th0 into Th17 and Th1, which may further exacerbate hypertension related immune dysfunction [29,30]. Previous animal studies also shown that Treg adoptive transfer could help to relive angiotensin II-induced hypertension [31] and aldosterone-induced endothelial dysfunction [32]. Those results indicated that, the association between hypertension and CD4+ T cells is mutually interactive and rather complex.

Although the role of CD4+ T cells has been widely discussed in hypertensive animal models, relevant epidemiological evidence remains limited. A clinical study including 45 patients with hypertension and 15 healthy subjects reported that compared with the healthy subjects, higher frequencies of circulating Th1 and Th17 and lower frequency of circulating Th2 were found in the hypertensive subjects [33]. Another prospective cohort study including 117 patients with connective tissue disease (CTD) and 53 patients with CTD-associated pulmonary arterial hypertension reported a significantly higher frequency of Th17 and higher level of circulating IL-17A in the patients with CTD-associated pulmonary arterial hypertension [34]. Partially in line with the previous studies, compared with the control group, significantly higher frequencies of Th1, Th1 (IFN-γ), and Th17 (IL-17A) were found in the participants with hypertension in this present study. In addition, a higher level of circulating IL-17A was also found in the case group. No statistical difference of on-site blood pressure was found between the two groups, which may be partially attributed to the significantly higher rate of antihypertensive drug use in the case group. It should be noted that a previous animal study found that blood pressure could be both ameliorated by Olmesartan and hydralazine; however, only Olmesartan could ameliorate the imbalance of T-cell subsets and attenuated renal injury induced by exogenous angiotensin II [35]. Furthermore, compared with the control group, a higher level of Treg was found in the participants with hypertension in this present study. Treg, with the secretion of anti-inflammatory IL-4, IL-10, and TGF-β, has been generally recognized as the crucial CD4+ T cell for the balance of systemic inflammation and relevant cardiovascular diseases [36]. Previous studies reported that the drugs of angiotensin II blocker [37] and aging [38] could increase the level of Treg. Those results indicated that, controlling blood pressure alone may not be enough to reverse the systematically inflammatory state in hypertensive patients; besides blood pressure control, anti-inflammation treatment should also be considered as an important goal in the treatment and management of hypertension.

### 4.2. Irrational Dietary Pattern, CD4+ T Cells and Hypertension

Over the past decade, many epidemiological studies have shown that obesity caused by a high-calorie, high-salt, high-fat diet was one of the most vital risk factors for hypertension. The DASH diet, which is characterized by a high consumption of fresh fruits, vegetables, and low-fat dairy products, is recommended as a rational dietary pattern for blood pressure control [9]. The geographical Jiangnan diet in China has also been recognized as a rational dietary pattern for the prevention and treatment of hypertension in China [10,11]. However, dietary patterns vary depending on depending on environment, climate, and food availability. For residents in northern China, especially those living in the rural area, there are several practical difficulties, such as eating habits and access to common food, to fully adopt the Jiangnan diet pattern.

In addition, although many epidemiological studies have confirmed the association between diet and hypertension, the mechanisms involved are not fully understood. Animal studies found that diets with high salt [39] or high fat [40] could both promote the expression of pro-inflammatory T-cell subsets, while the imbalance of T-cell subsets was a key target in the development of hypertension. Compared with the other subsets of CD4+ T cells, the pro-inflammatory Th17 seemed to be more suspectable to the components of the irrational dietary factors [41]. Several vitamins, including vitamin A [42] and vitamin D [43], were found to be effective to suppress Th17, which may be beneficial for the control of blood pressure. However, the association between the whole dietary pattern and Th cells in hypertension was rarely discussed. In this present study, one of the dietary patterns characterized by high factor loading of refined wheat, meat, poultry, and alcoholic beverage, was found to be positively associated with the increase of SBP (Table 5). Furthermore, this dietary pattern was adversely correlated with the anti-proinflammatory Th2 (IL-4). Meat, processed meat, and animal visceral showed statistically positive correlation with Th17 or Th1/17. Those results indicated that the irrational dietary pattern, characterized by a high consumption of animal foods, may be the independent risk factor for hypertension. In addition, Th17 may be involved in the association between dietary pattern and hypertension. However, this is limited evidence for the mechanisms underlying food consumption and CD4+ T cells; more studies needed to be conducted to analyzed the potential mechanism.

There were several limitations in this present study. The present study was a case-control study; more perspective studies were needed for the discussion of dietary pattern and hypertension. In addition, no rational dietary pattern was found to be recommended for the control of hypertension in this present study. It remained unclear whether modifying the existing dietary patterns could be effective in controlling hypertension in the rural area of northern China.

## 5. Conclusions

Five dietary patterns were identified in the middle-aged and elderly residents from the rural area of Beijing City, northern China. The irrational dietary pattern, characterized by a high consumption of refined wheat, meat, poultry, and alcoholic beverage, was positively associated with blood pressure. High circulating levels of pro-inflammatory CD4+ T cells were found in the patients with hypertension. Furthermore, meat, processed meat, animal visceral, and soft drink showed a statistically positive correlation with the pro-inflammatory Th17 or Th1/17. The increased risk of hypertension by irrational dietary pattern may be associated with Th17 cell.

## Figures and Tables

**Table 1:** General characteristics.

Characteristic	Control Group	Case Group	
	N = 56	N = 56	*p* Value
Male (*n*, %)	22 (39.3)	22 (39.3)	1.000
Age (y)	63 (57, 66)	63 (56.5, 66)	0.527
BMI (kg/m^2^)	26.0 (24.2, 28.1)	27.4 (24.7, 29.1)	0.411
WC (cm)	90 (84, 96)	92.5 (85, 98.5)	0.646
HC (cm)	97 (94, 103)	98.0 (95, 101.5)	0.851
HGS (kg)	26.8 (23.2, 33.6)	26.5 (22.0, 36.6)	0.971
Daily exercise level (*n*, %)			0.844
Light	38 (67.9)	36 (64.3)	
Moderate	6 (10.7)	8 (14.3)	
Heavy	12 (21.4)	12 (21.4)	
Daily salt consumption (*n*, %)			0.040
Light	13 (23.2)	24 (42.9)	
Moderate	21 (37.5)	11 (19.6)	
Heavy	22 (39.3)	21 (37.5)	
Daily fat consumption (*n*, %)			0.066
Light	10 (17.9)	21 (37.5)	
Moderate	26 (46.4)	19 (33.9)	
Heavy	20 (35.7)	16 (28.6)	
Current smoker (*n*, %)	11 (19.6)	7 (12.5)	0.303
Current drinker (*n*, %)	15 (26.8)	11 (19.6)	0.371
One-site SBP	134.3 (124.8, 149.3)	142.0 (127.5, 152.0)	0.365
One-site DBP	75.8 (70.5, 83.5)	80.3 (70.8, 88.0)	0.390
Antihypertensive drug use	21 (37.5)	36 (64.3)	0.005
T2Ds (*n*, %)	14 (25.0)	14 (25.0)	1.000
CKD (*n*, %)	5 (8.9)	5 (8.9)	1.000
Gout (*n*, %)	2 (3.6)	2 (3.6)	1.000
Hyperlipidemia (*n*, %)	19 (33.9)	18 (32.1)	0.841

Quantitative data are presented as median (Q1, Q3); categorical data are presented as *n* (%).

**Table 2:** Differences of CD4+ T-cell subsets in participants with and without hypertension.

CD4+ Subsets	Frequency (%)			Absolute Number (1000/mL)		
	Control Group	Case Group	*p* Value	Control Group	Case Group	*p* Value
Th0	32.0	27.3	0.009	280.5	210.6	0.195
	(21.4, 40.9)	(17.9, 34.5)		(161.3, 375.3)	(136.4, 353.8)	
Th1	20.6	24.5	0.001	171.3	220.6	0.020
	(15.7, 23.7)	(20.5, 28.6)		(119.9, 238.7)	(128.4, 303.3)	
Th2	14.4	15.5	0.438	122.0	123.0	0.607
	(11.6, 17.8)	(12.2, 18.7)		(88.1, 159.3)	(80.3, 197.0)	
Th17	13.7	15.2	0.687	118.8	133.5	0.448
	(11.7, 18.4)	(13.0, 17.4)		(99.0, 163.9)	(93.8, 176.0)	
Th1/17	17.1	16.9	0.955	122.7	138.8	0.448
	(13.1, 22.1)	(13.0, 21.7)		(101.4, 178.3)	(100.2, 208.3)	
Th1 (IFN-γ)	18.9	21.1	0.023	147.0	174.4	0.112
	(13.4, 25.3)	(17.2, 26.9)		(106.7, 203.6)	(125.4, 259.0)	
Th2 (IL-4)	1.6	1.5	0.664	12.0	11.5	0.695
	(1.1, 2.3)	(0.7, 2.5)		(8.4, 20.5)	(5.6, 21.5)	
Th17 (IL-17A)	1.4	1.7	0.027	10.4	12.7	0.091
	(1.1, 1.7)	(1.1, 2.3)		(7.7, 16.4)	(8.5, 21.2)	
Th1/17(IFN-γ/IL-17A)	0.3	0.4	0.003	1.9	2.4	0.017
	(0.1, 0.3)	(0.2, 0.6)		(1.0, 3.2)	(1.3, 5.4)	
Treg	2.6	4.0	<0.001	21.7	32.6	<0.001
	(1.9, 3.7)	(3.0, 4.7)		(18.1, 29.1)	(25.0, 40.9)	

**Table 3:** Comparison of inflammatory cytokines in the participants with and without hypertension.

Inflammatory Cytokines	Control Group	Case Group	*p* Value
IL-6 (pg/mL)	1.8 (0.9, 3.9)	1.0 (0.4, 2.2)	0.034
IL-10 (pg/mL)	10.5 (8.8, 12.6)	10.6 (7.9, 12.7)	0.660
IL-17A (pg/mL)	7.4 (5.3, 9.4)	8.2 (6.3, 10.3)	0.033

**Table 4:** Matrix factor loadings of all the food groups in the identified dietary patterns.

Food Group	DP1	DP2	DP3	DP4	DP5
Refined rice	0.19	0.32	−0.03	−0.18	0.16
Refined wheat	0.65	−0.15	−0.11	0.16	−0.05
Fried cereal	0.01	−0.02	−0.04	0.75	0.15
Coarse cereal	−0.02	0.23	−0.07	−0.28	0.40
Tuber	−0.21	0.56	0.05	0.02	0.11
Soybean	−0.13	0.70	−0.01	0.21	0.08
Legume	−0.02	0.76	−0.01	0.17	<0.01
Vegetables	0.18	0.43	0.09	−0.20	0.42
Mushroom and fungi	−0.05	0.26	−0.05	−0.04	−0.05
Pickles	0.19	0.11	−0.08	−0.02	0.37
Fermented food	0.06	0.30	−0.23	0.37	0.14
Fruit	−0.02	0.07	0.89	0.02	−0.06
Milk	−0.13	0.09	<0.01	−0.02	−0.20
Meat	0.78	0.01	0.10	0.10	−0.02
Poultry	0.76	−0.01	−0.05	0.04	0.22
Processed meat	−0.07	−0.07	0.86	0.02	0.13
Animal viscera	−0.09	−0.07	0.19	0.04	0.62
Fish and seafood	0.05	0.28	0.01	−0.15	−0.32
Eggs	0.01	0.31	0.11	0.52	−0.03
Nuts	0.06	0.39	0.12	−0.29	−0.21
Snacks	−0.01	0.19	0.01	0.16	0.48
Alcoholic beverage	0.73	−0.09	−0.05	−0.09	−0.04
Soft drink	0.15	−0.15	0.06	0.57	−0.07
Water	−0.32	−0.30	−0.12	−0.02	0.45
Explained variance	10.90%	10.12%	7.12%	6.97%	6.39%

**Table 5:** Association between dietary pattern and on-site blood pressure in multiple linear regression model.

DP	Model	On-Site SBP				On-Site DBP			
		*β*	95% CI		*p* Value	*β*	95% CI		*p* Value
DP1	1	3.96	0.25	7.66	0.037	2.29	−0.43	5.00	0.098
	2	5.38	0.73	10.03	0.024	3.47	−0.15	7.09	0.060
DP2	1	1.08	−2.70	4.86	0.572	0.89	−1.85	3.63	0.521
	2	1.27	−2.53	5.06	0.510	0.59	−2.35	3.52	0.692
DP3	1	−0.21	−3.99	3.57	0.912	−0.53	−3.27	2.22	0.704
	2	−1.04	−5.17	3.08	0.617	−0.64	−3.82	2.55	0.693
DP4	1	−1.99	−5.75	1.78	0.297	0.08	−2.67	2.82	0.957
	2	−3.76	−7.52	0.01	0.051	−0.62	−3.59	2.34	0.678
DP5	1	−0.52	−4.29	3.27	0.788	−0.27	−3.01	2.48	0.847
	2	0.17	−3.51	3.84	0.929	0.00	−2.84	2.84	1.000

Model 1: unadjusted model; Model 2: adjusted by age, gender, BMI, group, antihypertensive drug use, daily exercise level, T2Ds, CKD, gout, hyperlipidemia, smoke status, drink status, and self-reported dietary habits (salt and fat).

## Data Availability

Data are available from the corresponding author upon reasonable request due to privacy restrictions.

## Supplementary Materials

The following supporting information can be downloaded at: https://www.mdpi.com/article/10.3390/nu15020290/s1, Figure S1: Correlation between food group and the subset (absolute number) of CD4+ T cells.
