# Supplementary material for: The Increased Risk of Hypertension Caused by Irrational Dietary Pattern May Be Associated with Th17 Cell in the Middle-Aged and Elderly Rural Residents of Beijing City, Northern China: A 1:1 Matched Case-Control Study
Source: Nutrients. 2023 Jan 6;15(2):290. doi: 10.3390/nu15020290 (PMC9863205; doi:10.3390/nu15020290)
Supplement: Supplementary file 1 [file nutrients-15-00290-s001.zip › nutrients-2084796-supplementary.pdf]

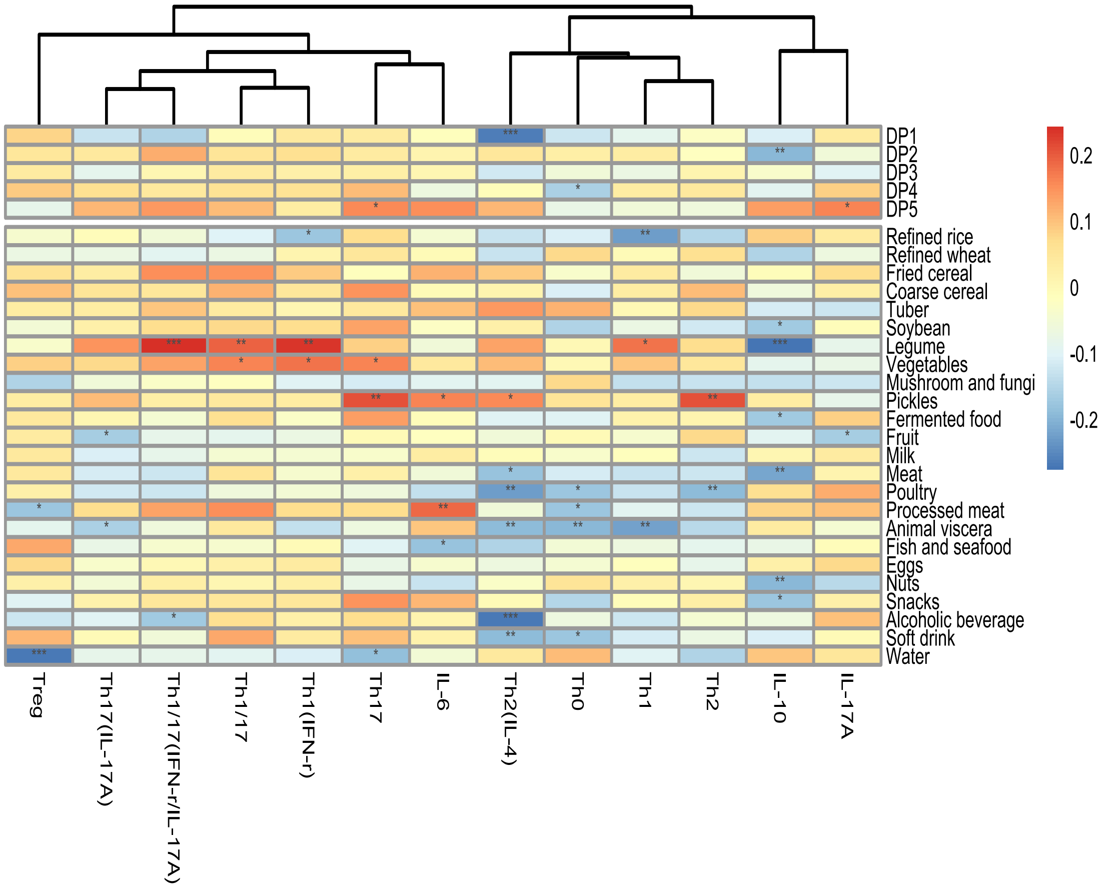

Supplementary Figure S1. Correlation between food group and the subset (absolute number) of CD4+ T cells. Correlation coefficient $r > 0$ was colored by red; $r < 0$ was colored by blue, * $p < 0.1$, ** $p < 0.05$, *** $p < 0.01$.
